# Chlamydia antibody testing helps in identifying females with possible tubal factor infertility

**Published:** 2016-03

**Authors:** Swapnil Singh, Shilpa Bhandari, Pallavi Agarwal, Priya Chittawar, Ratna Thakur

**Affiliations:** 1 *Department of Obstetrics and Gynaecology, Sri Aurobindo Medical College and PG Institute, Indore, India. *; 2 *Department of Reproductive Medicine, Sri Aurobindo Medical College and PG Institute, Indore, India. *

**Keywords:** *Sexually transmitted diseases*, *Chlamydial antibody*, *Laparoscopy*, *Infertility*, *Pelvic inflammatory disease*

## Abstract

**Introduction:**

Chlamydia is an important cause of sexually transmitted diseases leading to tubal factor infertility.

**Background:**

This study aims to define the role of chlamydial antibody detection in predicting presence, nature and type of tubal pathology in laparoscopy.

**Materials and Methods:**

A prospective study was conducted on 200 consecutive patients undergoing laparoscopy as a part of infertility work-up. Preoperatively, serological determination of Immunoglobulin G (IgG) specific antibodies against Chlamydia Trachomatis was done by Enzyme linked immunosorbant assay (ELISA). Findings of laparoscopy were evaluated against presence or absence of chlamydial antibodies in serum.

**Results:**

Out of 200 patients,10 patients tested positive for chlamydial antibody. Chlamydial antibody was found positive in 20% and 22.7% of patients with tubal pathology and peri-hepatic adhesions of patients, respectively. The sensitivity of chlamydial antibody for diagnosing tubal pathology was found to be 20%, while specificity was 100%. The positive chlamydial antibody test was not statistically associated with involvement of one or both tubes and site of tubal block.

**Conclusion:**

Chlamydia antibody test does not appear to be good screening test for tubal pathology especially in Indian subcontinent. In view of its high specificity, this test can be used to identify patients with higher chances of tubal pathology requiring operative intervention.

## Introduction

Infection with Chlamydia Trachomatis is an important cause of sexually transmitted disease world wide with extensive consequences for fertility resulting from damage to fallopian tube (1). Incidence of chlamydial infection as detailed in western literature is believed to be 4.2%, but data with regards to Indian subcontinent is lacking. In retrospective review, 2.2% of patients with infertility were found to be positive for chlamydia in cervical swabs (2, 3). 

In Indian subcontinent, tuberculosis and multi bacterial pelvic inflammatory disease are thought to be important causes of tubal damage leading to tubal factor infertility. The extent to which chlamydia is responsible for tubal factor infertility in Indian subcontinent is not clearly known. Association between chlamydia trachomatis antibody titres and tubal factor infertility has been known since 1979 and numerous studies have reported on value of chlamydia antibody titer (CAT) testing to predict tubal pathology (4). 

Pathogenic process of chlamydial infection is thought to be partly immunological and an association between C.Trachomatis heat shock protein 60 (HSP60) antibodies and sequel of infection has been observed (5). Sequel of this infection, namely PID is an important cause of tubal factor infertility. It has been observed that sequel is associated with persistent infection rather than single acute episode (6). 

Challenge faced with chlamydial disease is that as many as 70-80% infection is asymptomatic and diagnosis and identification of patients is hampered by lack of rapid, easy, sensitive and specific methods (7). Previous studies have shown that infertile women with tubal factor infertility are 2-4 times more likely to have elevated antibodies to chlamydia trachomatis than either infertile women with normal tubes or pregnant women, unlike HSG and laparoscopy, serological detection of chlamydia is non-invasive, simpler and faster to perform (8, 9).

The aim of present study is was to determine the association between tubal factor infertility and presence of chlamydial antibody. Furthermore, this study attempted to define the role of chlamydial antibody to predict tubal factor infertility in patients undergoing diagnostic laparoscopy.

## Materials and methods

this prospective study comprised 200 consecutive women scheduled for diagnostic laparoscopy as a part of infertility work-up from April 2013 to August 2014 in Department of Reproductive Medicine, Sri Aurobindo Medical College and PG Institute, Indore (India). Written informed consent was taken from each patient. Ethical clearance was taken from Sri Aurobindo Medical College and PG Institute Ethical Committee. 

Details of the patient’s age, type of infertility, duration of infertility, previously diagnosed pelvic infections were noted. Patients were evaluated preoperatively for their fitness to undergo laparoscopy after general medical history and blood investigations. Infertility was defined as failure to conceive after morethan a year of unprotected regular intercourse. Primary infertility was defined as a condition in which conception had never occurred, whereas term, secondary infertility was used to define those cases where there was an inability to conceive after previous successful conception. Laparoscopy was done in patients with suspected tubal factor infertility (abnormal HSG, history of pelvic surgery, endometriosis), unexplained infertility with previous failed IUI or those requiring operative procedures like myomectomy, cystectomy or ovarian drilling. Laparoscopy was performed postmenstrual in all patients using 3 punctures. Detailed examination of tubes and pelvic cavity was done and findings recorded. 

3 ml of venous blood sample was drawn preoperatively for laboratory measurement of serum IgG specific antibodies against chlamydia trachomatis by Enzyme inked Immunosorbent Assay (ELISA). The kits manual was strictly followed while tests were conducted. 

**Statistical analysis**

Analysis was done using Graphpad (Demo Version) software. ^2^ test was used to see statistical significant difference in distribution of discrete variables in two groups. Mann-Whitney U test was used to see the difference in mean of quantitative data in groups. P˂0.05 was considered significant. 

## Results

In our study, 200 infertile patients underwent chlamydia antibody testing and diagnostic laparoscopy. The demographic profile of patients enrolled in study is detailed in table I. In our study, only 5% (10/200) of women were seropositive for anti-chlamydial IgG antibody. There was no statistical difference in mean age of patients with positive and negative titres for chlamydial antibody (p=0.452). However, only 30% of patients with positive antibody titre had primary infertility in contrast to 64.73% with negative titres. Association of seropositivity with type of infertility appears to be statistically significant (p=0.0406) (Table I).

The positive predictive value of CAT test is 100%, while negative predictive value is 78.95% for diagnosing tubal disease. CAT test was positive in 10/50 patients of tubal disease so sensitivity was 20%, while the test had 100% specificity as it was negative in all 150 patients with normal tubes (Table II). Specificity of this test to diagnose perihepatic adhesions is 97.12%, while sensitivity is 22.73%, which is lower than that for tubal disease. The negative predictive value for perihepatic adhesions is high (91.05%) in comparison to positive predictive value, which is 50%.

The statistical association between tubal status and perihepatic adhesions with chlamydia antibody test appears to be significant (p=0.0004) while there appears to be non-significant association of this test with presence or absence of pelvic adhesions (p=0.5743) (Table II). Out of 200 patients who underwent laparoscopy, 50 (25%) were diagnosed with tubal disease. A total of 33 (66%) had bilateral tubal disease, whereas 17 (34%) had unilateral tubal pathology. 

Thirteen (6.5%) patients had multiple tubal pathology, either on same or both sides. Though majority of patients had bilateral tubal disease, this difference was not statistically significant with regards to seropositivity of chlamydial antibody (p=0.2768). Agglutinated fimbria (20/50, 40%) was most common tubal pathology noted in our series, whereas only 2 (4%) patients had isthmic block (Table III). The relation of site of tubal pathology is not associated with seropositivity of patients, although in seropositive patients agglutinated fimbria was the most common finding.

**Table I:** Patient profile and seropositivity (n=200)

	**Total **	**Seropositive (n=10)**	**Seronegative (n=190)**	**p-value**
Age (years)*	26.91 ± 3.49	28.1±4.28	26.85 ±3.45	0.452
Primary infertility#	126	3	123	0.0406
Secondary infertility#	74	7	67	

* Mann-Whitney U test was applied to see the significant difference in mean of age in two groups

# Chi-Square test was applied to see the significant difference in frequency of type of infertility in two groups

**Table II:** Cause of infertility and correlation with chlamydial seropositivity (n=200

	**Total**	**Seropositive (n=10)**	**Seronegative (n=190)**	**p-value** *
Tubal disease	50	10 (20%)	40 (80%)	<0.0001
Perihepatic adhesions	22	5 (22.73%)	17 (77.27%)	0.0004
Normal pelvic laparoscopy	144	0 (0)	144 (100%)	<0.0001
Pelvic adhesions	16	1 (6.25%)	15 ((93.75%)	0.5743

* Chi-Square test was applied to see the significant difference in frequency of different variables in two groups

**Table III.** Site of tubal block and chlamydial seropositivity

	**Total**	**Seropositive**	**Seronegative**	**p-value** *
Cornual	19	2	17	0.218
Ampullary	5	2	3	0.190
Fimbrial	20	5	15	0.494
Isthimic	2	0	2	1
Hydrosalpinx	12	3	9	0.686

* Chi-Square test was applied to see the significant difference in frequency of different variables in two groups

## Discussion

Our study aimed to define the role of chlamydia antibody test in predicting tubal pathology and its nature. In past, chlamydial infection has been more frequently associated in young females (age <20 years), but in our study, the mean age was 26.91±3.49 years (10). Our study population was infertile women which was not representative of general population. 

Also, previous literature pertains more to western civilization where onset of sexual activity is earlier in comparison to Indian sub-continent. In present study, seropositive status was seen in 3/126 (2.38%) patients with primary infertility in contrast to significantly higher proportion in patients with secondary infertility (9.46%). This is similar to previous reports, which hypothesized that higher titres may be related to increased risk factors for sexually transmitted infections, including increased numbers of sexual partners, in those with secondary infertility, or with higher prevalence of other causes of infertility (e.g., anovulation or endometriosis) in those with primary infertility (11). In previous Indian studies, 60-82.7% infertile female were found to be seropositive for chlamydia IgG antibodies (12-14). 

In present study only 5% of patients undergoing laparoscopy were found to be seropositive. Our results are different, possibly because our demographic profile is different too. Our study population comprised of only those women who underwent laparoscopy. Though the percentage of possible tubal factor infertility was higher, but other factors like unexplained, ovarian or uterine factor infertility were also parts of this cohort. The methodologies used to detect antibodies vary in their utility and populations studied may vary in their genetic predisposition to immune response and antibody production and persistence. Therefore, laboratory and regional differences could exist in chlamydial antibody testing.

In the present study, only 5% (10/200) of women had an IgG antibody titre in their blood signifying chlamydial infection while 25% (50/200) of women had evidence of tubal disease on laparoscopy. The sensitivity of chlamydia antibody test for detection of tubal disease was 20%. In a meta analysis, the sensitivity and specificity of this test varied between 21-90% and 29-100%, respectively (4). This variability is found to be subjected to how the tubal pathology was verified and type of chlamydia antibody titre assay. It was found that sensitivity of test increased if adhesions were not considered to be representative of tubal pathology. In present study, the patients belonged to region where tuberculosis is endemic. Therefore, it is possible that most of patients reporting with tubal factor infertility were more likely to be suffering from sequel of tuberculosis rather than chlamydia. 

The poor sensitivity of ELISA in present study is comparable to previous reports from Indian sub-continent (15). In this study, ELISA could detect on 3/100 cases from population attending sexually transmitted disease clinic. Thus, in spite of widespread availability, lower cost and ease of performance of ELISA, present study highlights its limitation to detect chlamydia induced tubal damage. Surana *et al* have also considered seropositivity for chlamydial antibody in relation to type and sites of tubal block (14). 

They found that seropositivity for chlamydia IgM antibody was the highest among the subjects with a fimbrial blockage (80%), followed by those with an ampullary blockage (66.6%). This is similar to our study even though it was observed that this association was not statistically significant. Higher incidence of agglutinated fimbria and fimbrial block in seropositive cases suggests that chlamydial infection is associated with peripheral endosalpingitis. This has been confirmed by findings of previous studies as well (16).

Surana *et al* also demonstrated a significant association between seropositivity and bilateral tubal disease (14). In our study, even though bilateral tubal disease was observed in 2/3 of patients, the association with seropositivity was not significant. Unilateral tubal disease may compromise fertility prospects moderately as against bilateral tubal disease. Unfortunately, CAT test does not predict involvement of single or both tubes. Therefore, as detection of unilateral tubal disease is unlikely to result in a major change in treatment, laparoscopy is still required to plan and prognosticate further treatment.

In present study, positive chlamydia antibody test was found to be a statistically significant predictor of tubal pathology. In a similar study, out of 21 patients with tubal factor infertility, 20 had positive titres, which was significantly higher in comparison with their fertile controls (9). This fact can be utilized to construct a triage such that patient with a positive CAT test are subjected to laparoscopy earlier. Coppus*et al* have evaluated the efficiency of a combination of medical history and CAT testing in selecting women for laparoscopy to detect tubal pathology (17).

They found that combined interpretation of both identified women at the highest risk for tubal disease while in cases with negative CAT status and non-suspect clinical history laparoscopy could be deferred.

**Limitations:**

First, this study has not considered other causes of tubal pathology, which could have explained the lower sensitivity of CAT test. Second limitation is that the cohort of women with positive CAT test is very small. But as decision of laparoscopy was taken irrespective of CAT test result, the diagnostic performance can be estimated without partial verification bias. Third limitation is that nature of tubal disease and its impact on fertility has not been considered. 

One of the most important advantages of laparoscopy is that it not only diagnoses tubal pathology, but also provides an opportunity to correct it and increase the fertility potential of women. The present study does not answer whether the tubal pathology predicted by a positive CAT test is amenable to correction or its impact on fecundity.

## Conclusion

Our study demonstrates that chlamydia antibody test is very specific for tubal disease detection, even though the sensitivity is low. Therefore, this is not a good screening tool for tubal factor infertility, especially in Indian sub-continent. We believe that chlamydia antibody test predicts the presence of tubal pathology with high accuracy, but does not define its impact on fecundity. Therefore, this test can only be used to identify patients with tubal pathology requiring operative laparoscopy.
